# Isoform-specific Inhibition of *N*-methyl-D-aspartate Receptors by Bile Salts

**DOI:** 10.1038/s41598-019-46496-y

**Published:** 2019-07-11

**Authors:** Angela Koch, Michele Bonus, Holger Gohlke, Nikolaj Klöcker

**Affiliations:** 1 Institute of Neural and Sensory Physiology, Medical Faculty, Heinrich Heine University Düsseldorf, 40225 Düsseldorf, Germany; 2 Institute for Pharmaceutical and Medicinal Chemistry, Heinrich Heine University Düsseldorf, 40225 Düsseldorf, Germany; 3 John von Neumann Institute for Computing (NIC), Jülich Supercomputing Centre (JSC) & Institute for Complex Systems - Structural Biochemistry (ICS 6), Forschungszentrum Jülich GmbH, 52425 Jülich, Germany

**Keywords:** Physiology, Gastroenterology

## Abstract

The *N*-methyl-D-aspartate subfamily of ionotropic glutamate receptors (NMDARs) is well known for its important roles in the central nervous system (CNS), e.g. learning and memory formation. Besides the CNS, NMDARs are also expressed in numerous peripheral tissues including the pancreas, kidney, stomach, and blood cells, where an understanding of their physiological and pathophysiological roles is only evolving. Whereas subunit composition increases functional diversity of NMDARs, a great number of endogenous cues tune receptor signaling. Here, we characterized the effects of the steroid bile salts cholate and chenodeoxycholate (CDC) on recombinantly expressed NMDARs of defined molecular composition. CDC inhibited NMDARs in an isoform-dependent manner, preferring GluN2D and GluN3B over GluN2A and GluN2B receptors. Determined IC_50_ values were in the range of bile salt serum concentrations in severe cholestatic disease states, pointing at a putative pathophysiological significance of the identified receptor modulation. Both pharmacological and molecular simulation analyses indicate that CDC acts allosterically on GluN2D, whereas it competes with agonist binding on GluN3B receptors. Such differential modes of inhibition may allow isoform-specific targeted interference with the NMDAR/bile salt interaction. In summary, our study provides further molecular insight into the modulation of NMDARs by endogenous steroids and points at a putative pathophysiological role of the receptors in cholestatic disease.

## Introduction

*N*-methyl-D-aspartate receptors (NMDARs) comprise a subfamily of ionotropic glutamate receptors (iGluRs). NMDARs assemble from combinations of GluN1, GluN2 and GluN3 subunits, whereby they build tetrameric ion channels, with GluN1 being obligatory for channel function. Distinct from other iGluR subfamilies, most NMDARs show significant Ca^2+^ permeability and exhibit an apparent voltage-dependence due to voltage-dependent block by extracellular Mg^2+^ ions^1,2^. Activation of most NMDAR subunit combinations requires binding of the agonist glutamate to GluN2 and the co-agonists glycine or D-serine to GluN1 subunits^3^. The exceptions are channels assembled from GluN1 and GluN3, which lack a glutamate-binding site and hence serve as glycine-activated cation channels^4^. Aside from their well-characterized expression in the brain, both in neuronal and non-neuronal cells, NMDARs have been identified in a number of peripheral tissues and systems^5^. These include circulating blood cells such as lymphocytes and platelets, which may employ NMDARs for cell activation. As non-neuronal NMDARs are presumably directly exposed to high levels of plasma glutamate, alternative agonists such as L-homocysteic acid (L-HCA) and quinolinic acid (QN) are discussed as receptor ligands^5^.

NMDAR activity is modulated by numerous endogenous cues including pH, metal ions, polyamines, and lipids^6^. Neurosteroids derived from the cholesterol backbone may allosterically either potentiate or inhibit NMDAR function in an isoform-dependent manner^6–9^. The main steroid salts contained in human bile, cholate and chenodeoxycholate (CDC), are also derived from cholesterol. These primary bile salts are synthesized by hepatocytes, conjugated to glycine or taurine to increase water solubility, and finally secreted into the intestine to help emulsifying food lipids. Bile salts are efficiently reabsorbed from the intestine and transported back to the liver for reuse by the hepatic portal system (enterohepatic cycle). Under physiological conditions, only insignificant amounts of bile salts enter systemic circulation. In certain cholestastic disease states like progressive familial forms of intrahepatic cholestasis or severe forms of it in pregnancy or acute hepatitis, however, plasma concentrations of bile salts may rise to hundred µM and more^10–12^. When applied to neurons, cholate and CDC block NMDA receptors^13^ and it is known that CDC can improve neurological symptoms of cerebrotendinous xanthomatosis, a disorder of bile salt metabolism^14^. Another bile salt, tauroursodeoxycholate, is neuroprotective on NMDA-induced retinal ganglion cell degeneration^15^. Furthermore, NMDA receptors are reported to be involved in modulation of cholestasis-induced antinociception in rat^16,17^ and there is a potential involvement of the dorsal hippocampal (CA1) glutamatergic system in cholestasis-induced decrease in rats’ memory function^18^. Thus, bile salt effects on NMDA receptors can have a physiological and pathophysiological relevance in nervous system or peripheral tissues.

Here, we have studied the effects of the most common human bile salts, cholate and CDC, on NMDARs of different subunit composition. We demonstrate that bile salts inhibit NMDARs in an isoform-specific manner, both with respect to efficacy and to the molecular mechanism of action. Our data shed new light on the effects of bile salts in cholestatic diseases as well as when used in therapy.

## Results

### CDC inhibits preferentially the GluN subtypes 2D and 3B

**Figure 1:**
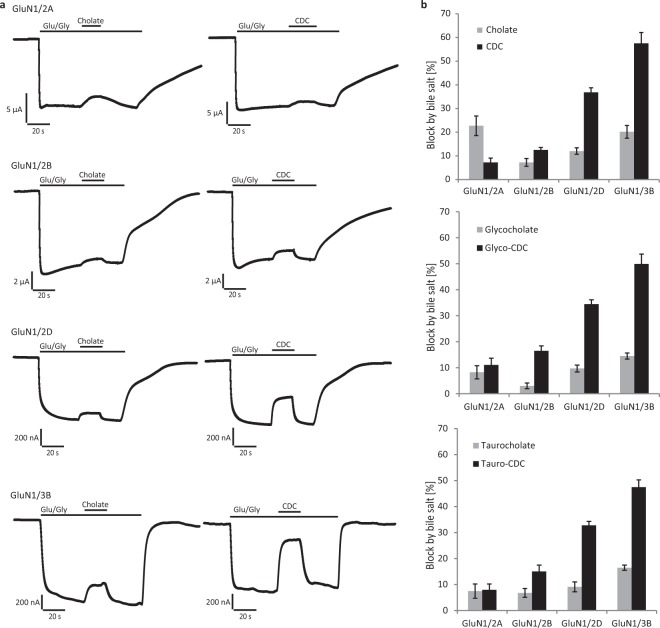
Inhibition of NMDARs by the bile salts cholate and CDC. (**a**) Representative current traces recorded from *Xenopus laevis* oocytes expressing NMDARs of indicated molecular compositions. Note the reversible reduction of agonist-induced currents by cholate and CDC. (**b**) Quantification of the experiments in (**a**). Given is the mean reduction (±SEM) of indicated NMDAR currents by application of 100 μM bile salts (in % of steady-state agonist-induced current, n = 6–14 oocytes). Glutamate (Glu): 150 µM, Glycine (Gly): 10 µM.

The effects of the bile salts cholate and CDC on GluNs were probed on recombinant receptors of defined subunit composition expressed in *Xenopus laevis* oocytes. For initial experiments, agonists were applied at saturating concentration to ensure maximum activation of GluN2A, GluN2B, GluN2D, and GluN3B, respectively^4,19^. Cholate inhibited steady-state currents only moderately irrespective of receptor subtype, while CDC was more potent and reduced preferentially GluN2D and GluN3B currents (Fig. 1, Table S1). Inhibition by 100 µM CDC ranged between 7 ± 2% of GluN2A and 13 ± 1% of GluN2B steady-state currents, whereas it amounted to 37 ± 2% of GluN2D and even to 58 ± 5% of GluN3B currents. The bile salt effects were specific for GluNs, as no inhibition of glutamate-induced steady-state kainate receptor GluK2 current (n = 3, after block of desensitization with ConA) and only a minor reduction of α-amino-3-hydroxy-5-methyl-4-isoxazolepropionic acid (AMPA) receptor GluA1/GluA2 currents (7 ± 1%, n = 6) were observed after application of 100 µM CDC. Somewhat unexpected, taurine or glycine conjugation of bile salts did neither change their overall potencies in inhibiting GluNs nor their subunit preferences.

### Allosteric inhibition of GluN2D by tauro-CDC

To dissect the mechanism of GluN2D inhibition by bile salts, we recorded dose-response relations for CDC at both saturating and EC_50_ NMDAR agonist concentrations^20,21^. We used routinely taurine-conjugated CDC in these experiments, because of higher water solubility and hence more feasible handling.

**Figure 2:**
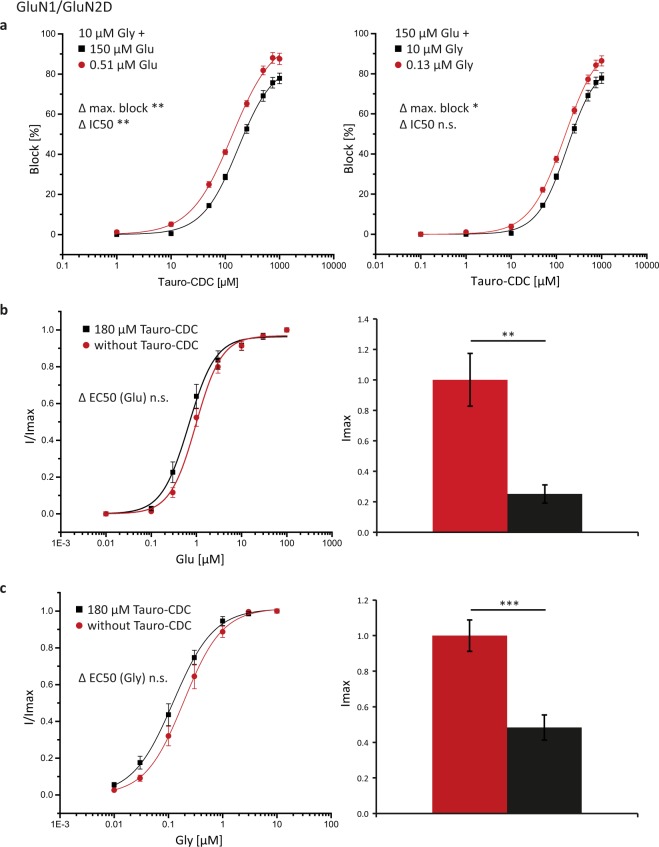
Tauro-CDC inhibits GluN2D receptors in an allosteric manner. (**a**) Concentration-response curves for inhibition of GluN1/GluN2D receptors by tauro-CDC at saturating (squares) and half-maximum (dots) agonist concentrations (glutamate/glycine, Glu/Gly) as indicated^20,21^. Displayed is mean ± SEM of recordings from n = 8–13 oocytes. (**b**) Concentration-response curves (left) and maximum currents (right) induced by the agonist Glu in the presence (squares) and absence (dots) of 180 µM tauro-CDC. The concentration of the co-agonist Gly was kept constant at 10 µM. Displayed is the mean ± SEM of recordings from n = 7 oocytes. (**c**) Concentration-response curves and maximum currents induced by the co-agonist Gly in the presence (squares) and absence (dots) of 180 µM tauro-CDC. The concentration of the agonist Glu was kept constant at 150 µM. Displayed is the mean ± SEM of recordings from n = 12–13 oocytes. The results of t-tests are reported as follows: n.s. p > 0.05, *p ≤ 0.05, **p ≤ 0.01, ***p < 0.001.

As depicted in Fig. 2a, maximum inhibition of glutamate/glycine-induced currents by tauro-CDC was significantly smaller at saturating than at EC_50_ agonist concentrations (n = 8–13; p = 0.01 and 0.03 for glutamate and glycine, respectively).

Conversely, dose-response curves for glutamate and glycine in the absence or presence of 180 μM tauro-CDC, which was calculated to be the mean IC_50_ (179 ± 11 μM, n = 13, saturating agonist concentration), showed that the maximum agonist-induced currents were significantly reduced by the bile salt (Fig. 2b,c; n = 7–13, p = 0.0002 and 0.003 for glycine and glutamate, respectively). There were neither significant differences between the EC_50_ values for glutamate or glycine at varying concentrations of tauro-CDC (n = 7–13, p = 0.3 and 0.2 for glutamate and glycine respectively) nor were there different IC_50_ values for tauro-CDC at varying concentrations of glycine (n = 8–13; p = 0.1; Table S2). Only the IC_50_ value for tauro-CDC at the glutamate EC_50_ concentration was significantly smaller compared to inhibition at a saturating glutamate concentration (n = 11–13, p = 0.009).

Application of 100 µM tauro-CDC in the absence of NMDAR agonists showed no effects on GluN2D or GluN3B receptors (n = 4) excluding partial agonist action of bile salts on NMDA receptors (data not shown). Taken together, these results strongly suggest that tauro-CDC inhibits GluN2D receptors by an allosteric mechanism.

### Competitive inhibition of GluN3B by tauro-CDC

**Figure 3:**
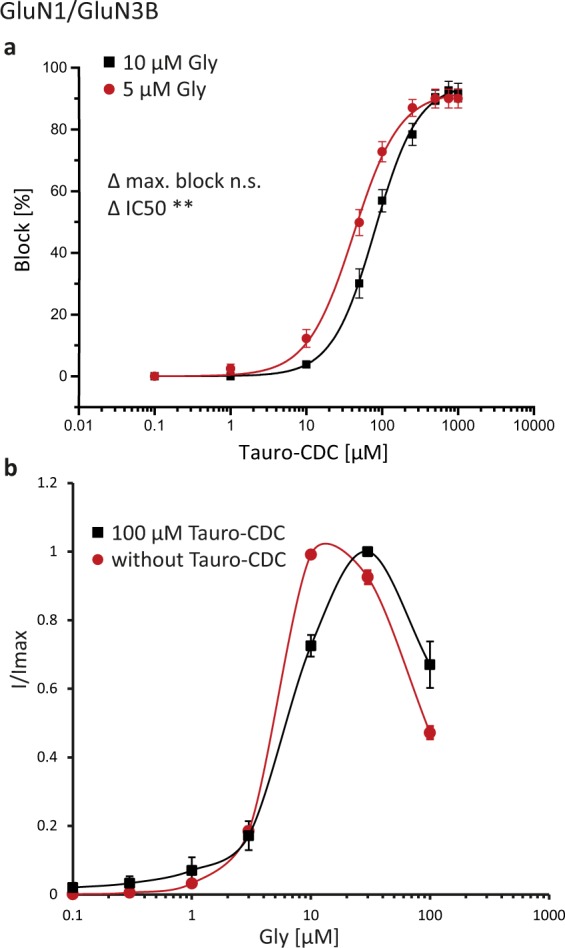
Tauro-CDC inhibits GluN3B receptors in a competitive manner. (**a**) Concentration-response curves for inhibition of GluN1/GluN3B receptors by tauro-CDC at saturating (squares) and estimated half-maximum (dots) glycine (Gly) concentrations as indicated^4^. Displayed is mean ± SEM of recordings from n = 6 oocytes. (**b**) Concentration-response curves for Gly in the presence or absence of 100 μM Tauro-CDC (n = 6–11 oocytes). The results of t-tests are reported as follows: n.s. p > 0.05, *p ≤ 0.05, **p ≤ 0.01, ***p < 0.001.

Next, we sought to investigate the mechanism of action of tauro-CDC-mediated inhibition of GluN3B receptors. As depicted in Fig. 3a, the mean IC_50_ for tauro-CDC was significantly higher in the presence of 10 μM glycine than in the presence of 5 μM glycine (83 ± 8 μM vs. 44 ± 7 μM, n = 6, p = 0.007). The maximum block by tauro-CDC, however, did not depend on the glycine concentration (95 ± 3% vs. 92 ± 3%, n = 6, p = 0.5). These data indicate that tauro-CDC inhibits GluN3B receptors – other than GluN2D receptors – in a competitive manner. We also recorded glycine dose-response curves in the presence or absence of 100 μM tauro-CDC (n = 6–11, Fig. 3b). Known receptor desensitization at higher glycine concentrations prevented calculation of precise EC_50_ values^4,22^. However, as depicted in Fig. 3b, the bell-shaped concentration-response curve shifted to higher glycine concentrations in the presence of tauro-CDC.

### Tauro-CDC has no effect on ion selectivity

**Figure 4:**
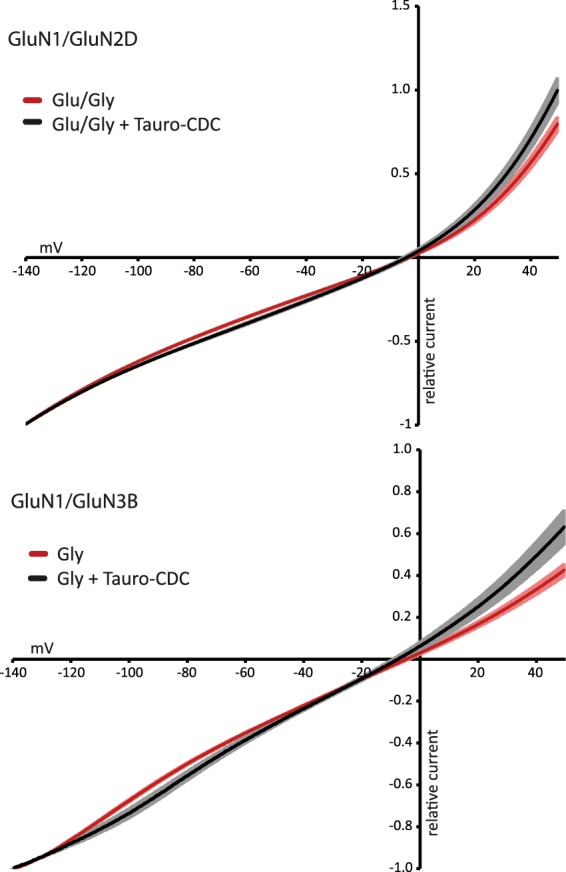
Tauro-CDC does not affect the ion selectivity of NMDARs. I/V relationships (n = 10–11, mean ± SEM) for indicated NMDARs of agonist-induced currents in presence (black) and absence (red) of 100 μM tauro-CDC. Data were normalized to maximum inward current at –140 mV. Note that the reversal potentials do not change in the presence of tauro-CDC.

Bile salts are known to integrate into the plasma membrane of cells^23^, which might impact ion channel pores including their selectivity filters. Therefore, we checked for potential bile salt effects on GluN receptor ion selectivity. Current-voltage (I/V) relationships of agonist-induced currents were recorded for GluN2D and GluN3B in the absence and presence of 100 µM tauro-CDC (n = 10–11). For better comparison of I/V curves, individual oocyte data were normalized to their maximum inward current at −140 mV. As shown in Fig. 4, tauro-CDC did neither change the shape of I/V relationships nor did it shift the reversal potentials of the receptor currents. These results render a change in ion selectivity of the two GluNs by bile salts unlikely.

### Bile salt inhibition of GluNs when activated by alternative agonists

As the inhibition of GluNs by bile salts required concentrations that are hardly reached in the CNS, we tested whether tauro-CDC also reduced currents elicited by the alternative non-neuronal NMDAR agonists L-homocysteic acid (L-HCA) and quinolinic acid (QN)^5^.

**Figure 5:**
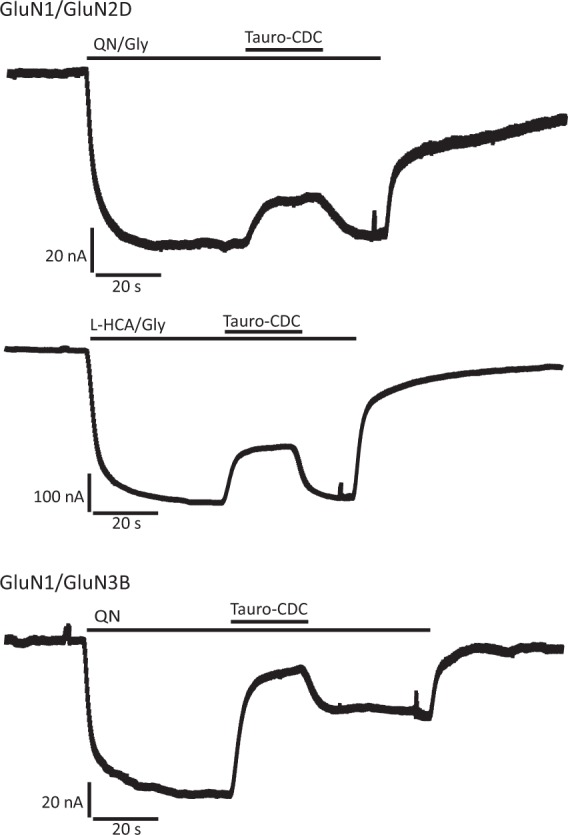
Bile salt inhibition of NMDARs activated by the alternative agonists quinolinic acid (QN) and L-homocysteic acid (L-HCA). Representative current traces recorded from *Xenopus laevis* oocytes expressing NMDARs of indicated molecular compositions. Note the reversible reduction of currents induced by QN (7.2 mM) and L-HCA (13 µM) by tauro-CDC (100 µM). Concentrations of QN and L-HCA are reported EC_50_^24^.

As shown in Fig. 5, GluN2D currents induced by application of L-HCA at its reported EC_50_ of 13 µM^24^ were blocked by 30 ± 2% (n = 12), whereas currents elicited by QN at its reported EC_50_ of 7.2 mM^24^ were reduced by 26 ± 2% (n = 7) when co-applying tauro-CDC at 100 µM. Lowering the agonist concentration of QN to 1 mM did not change inhibition by tauro-CDC, which was still 29 ± 2% (n = 7). For all these experiments involving the non-neuronal NMDAR agonists L-HCA and QN on GluN2D receptors, 10 µM glycine was used as co-agonist. GluN3B could not be activated by L-HCA, even at high concentrations of up to 1 mM (n = 4). In contrast, currents activated by 7.2 mM QN were reduced by about 68 ± 4% (n = 5), with QN showing no effect on uninjected oocytes (n = 3).

### Binding modes of tauro-CDC in GluN2D_LBD_ and GluN3B_LBD_

**Figure 6:**
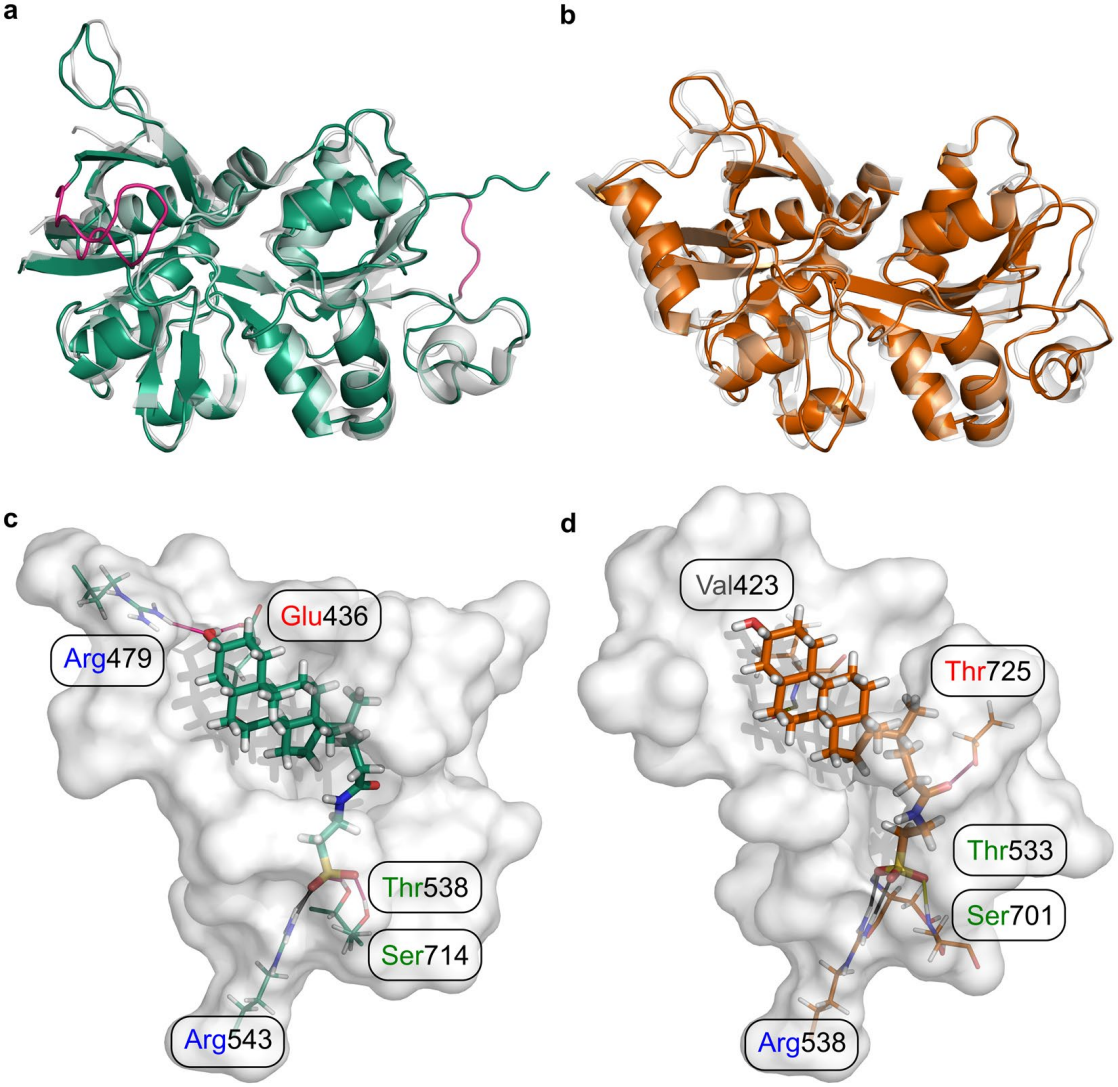
Homology models of GluN2D_LBD_ and GluN3B_LBD_ and docking-derived complex structures with tauro-CDC. (**a**) Homology model of GluN2D_LBD_ (green). The loop consisting of residues 464–476 (magenta, left) was modeled *de novo* due to its absence in the template structure (grey, PDB ID: 3OEK^45^). The engineered Gly-Thr linker in the template structure (magenta, right) was removed in the final model. (**b**) Homology model of GluN2D_LBD_ (orange) superimposed onto the template structure (grey, PDB ID: 2RCA^32^). (**c**) Binding mode of tauro-CDC (green sticks) in GluN2D_LBD_ (white surface) predicted by docking. Residues involved in hydrogen bonds and salt bridges are depicted as lines. Positively and negatively charged residues are labeled in blue and red, respectively. Polar residues are labeled in green. (**d**) Binding mode of tauro-CDC (orange sticks) in GluN3B_LBD_ (white surface) predicted by docking. Residues involved in hydrogen bonds and salt bridges are depicted as lines. Positively and negatively charged residues are labeled in blue and red, respectively. Polar residues are labeled in green and hydrophobic residues are labeled in grey.

The pharmacological experiments indicated that tauro-CDC acts as a non-competitive allosteric antagonist of GluN2D, but as a competitive antagonist of GluN3B. In order to assess the molecular origin of isoform-specific inhibition of NMDARs by tauro-CDC, we performed induced fit docking experiments of tauro-CDC in respective ligand binding domains (LBDs) of the NMDAR isoforms (Fig. 6a,b), which allows to consider receptor flexibility in terms of side chain rearrangements.

For both GluN2D_LBD_ and GluN3B_LBD_, binding modes of tauro-CDC (Fig. 6c,d) were predicted with similar docking scores (IFDScore^25^) of ~7.4 kcal·mol^−1^. In agreement, both binding modes were of similar geometry with an in-place root-mean-square deviation (RMSD) of the ligand atoms of 2.98 Å after superimposition of the C_α_ atoms of the protein structures (RMSD: 1.45 Å). The similarity in binding mode geometry was paralleled by a similar interaction profile in both complexes (Fig. 6c,d).

Hence, the static structures obtained from docking experiments cannot explain the observed isoform specificity of NMDAR inhibition by tauro-CDC.

### Structural variability of tauro-CDC/GluN2D_LBD_ and tauro-CDC/GluN3B_LBD_ binding modes

We next asked whether the predicted binding modes of tauro-CDC in GluN2D_LBD_ or GluN3B_LBD_ structurally vary over time scales accessible by molecular dynamics (MD) simulations. We therefore carried out ten MD simulations (S_1_-S_10_) of 500 ns length each for both the tauro-CDC/GluN2D_LBD_ and tauro-CDC/GluN3B_LBD_ systems and quantified the structural deviation from the initial tauro-CDC pose within each simulation and between independent simulations. Similarly, we quantified the conformational variability of tauro-CDC to account for potential alternative binding modes.

Taking all simulations but S_9_ into account, the average RMSD of the tauro-CDC pose in GluN2D_LBD_ was 2.86 ± 0.35 Å higher than in GluN3B_LBD_ (Supplementary Fig. 3a). Simulation S_9_ of GluN2D_LBD_ was omitted from this and all subsequent analyses when tauro-CDC unbound after ~225 ns (Supplementary Fig. 3b). No unbinding event was observed in the simulations of tauro-CDC/GluN3B_LBD_.

**Figure 7:**
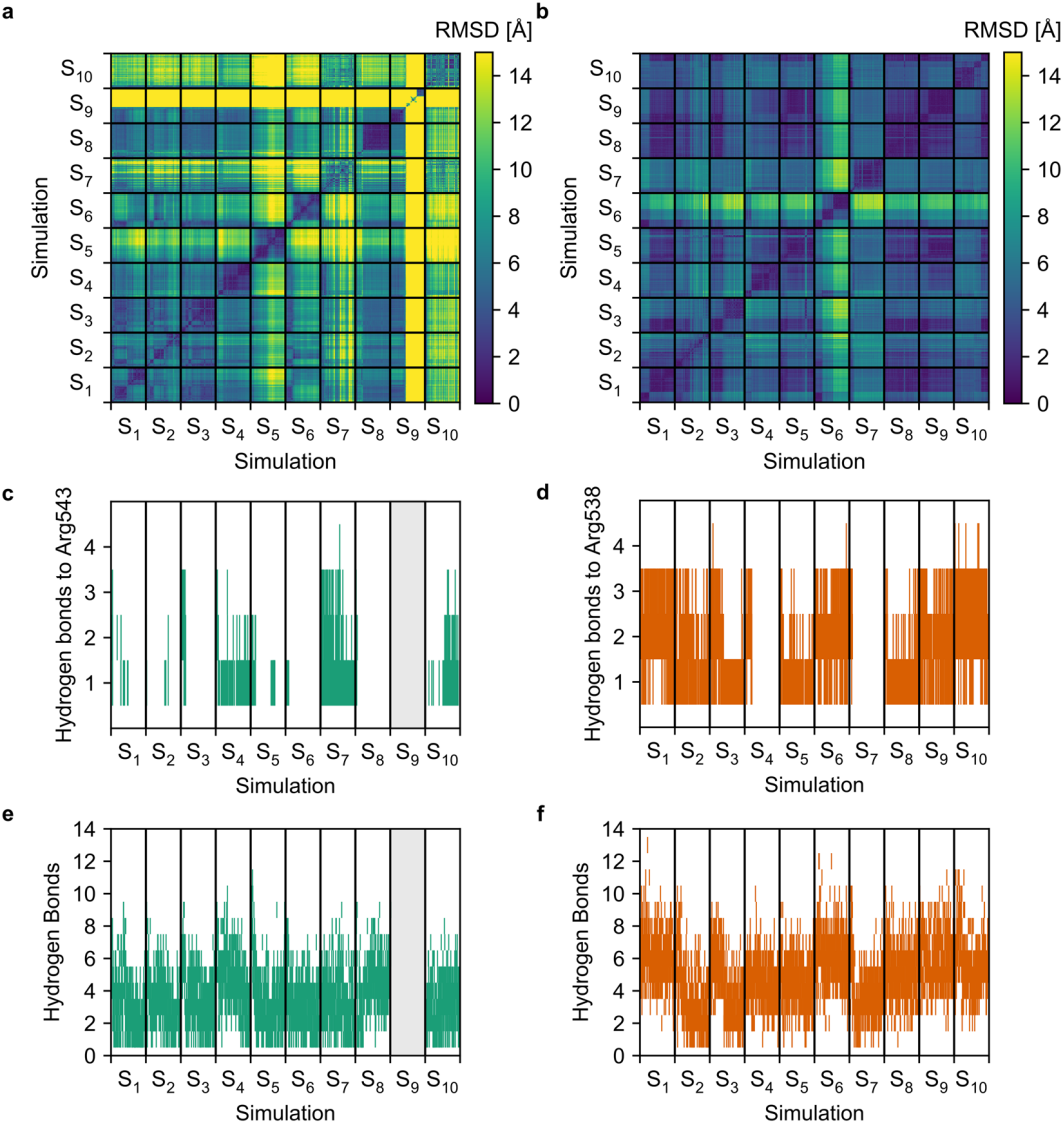
Structural variability and number of hydrogen bonds in the tauro-CDC-GluN2D_LBD_ and tauro-CDC-GluN3B_LBD_ complexes during the MD simulations. (**a**) Two-dimensional root-mean-square deviation (RMSD) of the atomic positions of tauro-CDC after least squares fitting of the C_α_ atom coordinates of the GluN2D_LDB_ in all 10 MD simulations. (**b**) Two-dimensional root-mean-square deviation (RMSD) of the atomic positions of tauro-CDC after least squares fitting of the C_α_ atom coordinates of the GluN3B_LDB_ in all 10 MD simulations. (**c**) Number of hydrogen bonds between tauro-CDC and Arg543 in GluN2D_LBD_ over the course of 10 MD simulations. (**d**) Number of hydrogen bonds between tauro-CDC and Arg538 in GluN3B_LBD_ over the course of 10 MD simulations. (**e**) Total humber of hydrogen bonds between tauro-CDC and GluN2D_LBD_ over the course of 10 MD simulations. (**f**) Total number of hydrogen bonds between tauro-CDC and GluN3B_LBD_ over the course of 10 MD simulations. In panels c and e, data for MD simulation S_9_ is not shown because here tauro-CDC unbinds after 225 ns (c.f. Supplementary Fig. 3b), and the corresponding data was excluded from statistical analysis.

The average pairwise RMSD of the tauro-CDC pose of all snapshots extracted from the MD simulations was 8.44 ± 0.001 Å for tauro-CDC/GluN2D_LBD_ (Figs. 7a) and 4.82 ± 0.001 Å for tauro-CDC/GluN3B_LBD_ (Fig. 7b). Taking the quadratic mean of the pairwise RMSD as a measure for pose diversity^26^ revealed that the tauro-CDC poses observed both within and between simulations were substantially more diverse in GluN2D_LBD_ systems (mean: 8.43 ± 0.46 Å, Supplementary Fig. 3c) than in GluN3B_LBD_ systems (mean: 4.89 ± 0.26 Å, Supplementary Fig. 3d) (*t*(98) = 7.01, *p* < 10^–4^).

To conclude, these results reveal that the predicted binding mode of tauro-CDC in GluN2D_LBD_ is structurally less stable than in GluN3B_LBD_. In one simulation of tauro-CDC in GluN2D_LBD_, tauro-CDC even unbound. Note that simulating ligand unbinding events is challenging also for ligands with moderate affinities due to low off-rates^27^, which may explain why the unbinding occurred only in one simulation. As to GluN3B_LBD_, the results are in line with our experimental data, according to which tauro-CDC must bind to the glycine binding site in order to competitively inhibit GluN3B-containing NMDARs.

### Contributions of hydrogen bonds to tauro-CDC/GluN2D_LBD_ and tauro-CDC/GluN3B_LBD_ complex stability

To assess whether a difference in structural variability between tauro-CDC/GluN2D_LBD_ and tauro-CDC/GluN3B_LBD_ may be attributed to differences in intermolecular interactions within the complexes, we quantified the amount of hydrogen bonds formed between tauro-CDC and an arginine residue that is crucial for binding of the agonist glutamate to GluN2D_LBD_ (Arg543) and of glycine to GluN3B_LBD_ (Arg538) over the course of all MD simulations. Additionally, we quantified the total amount of hydrogen bonds between tauro-CDC and GluN2D_LBD_ or GluN3B_LBD_. We focused here on identifying hydrogen bonds as interactions that contribute to the stability of the tauro-CDC/GluN3B_LBD_ complex in order to predict mutations for *ex vivo* validation of a suggested tauro-CDC binding mode.

The average hydrogen bond count between Arg543 in GluN2D_LBD_ and tauro-CDC was 0.31 ± 0.01 (Fig. 7c). In contrast, Arg538 in GluN3B_LBD_ formed hydrogen bonds to tauro-CDC about four times more frequently (mean: 1.31 ± 0.01) (*t*(9498) = 58.58, *p* < 10^−4^) (Fig. 7d). Similarly, the average number of hydrogen bonds formed between the complete GluN2D_LBD_ and tauro-CDC (mean: 3.58 ± 0.03) (Fig. 7e) was significantly lower than the average number of hydrogen bonds formed between the complete GluN3B_LBD_ and tauro-CDC (mean: 4.97 ± 0.03) (*t*(9498) = 38.10, *p* < 10^−4^) (Fig. 7f).

**Figure 8:**
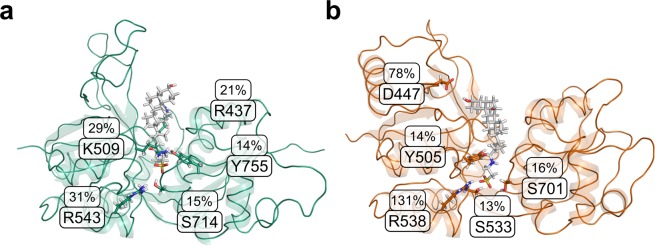
Formation of protein-ligand hydrogen bonds during the MD simulations. Average hydrogen bond occupancy of the five amino acids most frequently involved in hydrogen bonding to tauro-CDC in (**a**) the 9 tauro-CDC-GluN2D_LBD_ simulations and (**b**) the 10 tauro-CDC-GluN3B_LBD_ simulations.

At a per-residue level, Arg543 showed the largest hydrogen bond occupancy (31%) for tauro-CDC/GluN2D_LBD_ (Fig. 8a). The corresponding residue in GluN3B_LBD_ (Arg538) displayed an occupancy of 131% (occupancy may exceed 100% as an arginine side chain can form more than one hydrogen bond) (Fig. 8b). Tauro-CDC in GluN3B_LBD_ also formed a hydrogen bond with the 3α-OH group of the cholane scaffold to Asp447 (78% occupancy). This residue has no counterpart in the GluN2D sequence (Supplementary Fig. 1). In addition to Arg538 and Asp447, Ser701 (16%), Tyr505 (14%), and Ser533 (13%) were among the five residues that most frequently formed hydrogen bonds to tauro-CDC during the GluN3B_LBD_ simulations (Fig. 8b). Lys509 (29%), Arg437 (21%), Ser714 (15%) and Tyr755 (14%) additionally stabilized the GluN2D_LBD_-tauro-CDC complex.

To conclude, hydrogen bond formation between tauro-CDC and Arg543 in GluN2D_LBD_ occurred less frequently than between tauro-CDC and Arg538 in GluN3B_LBD_; the same held true when considering hydrogen bond formation to the complete LBD. The tauro-CDC/GluN3B_LBD_ complex was particularly stabilized by Arg538 and, to a lesser extent, Asp447, whereas no particular amino acid contributed to stabilizing tauro-CDC/GluN2D_LBD_.

### Effective binding energies of tauro-CDC/GluN2D_LBD_ and tauro-CDC/GluN3B_LBD_ complexes

To complement our findings from the structural analysis of tauro-CDC complexes, we quantified the effective binding energy of tauro-CDC to GluN2D_LBD_ or GluN3B_LBD_ with the end-point effective energy approaches MM/PBSA and MM/GBSA.

**Figure 9:**
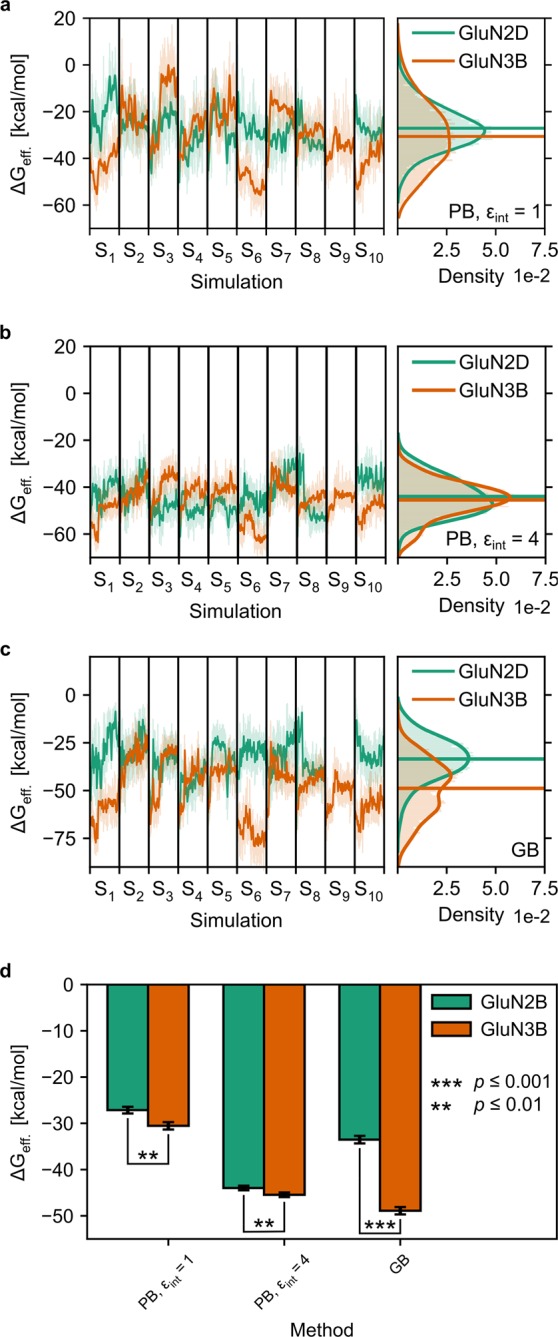
Effective binding energies (Δ*G*_eff_) of the tauro-CDC-GluN2D_LBD_ (green) and tauro-CDC-GluN3B_LBD_ (orange) complexes. (**a**–**c**) Time courses (left) and probability distributions (right) of Δ*G*_eff_, calculated using either the MM-PBSA method and an internal dielectric constant of (**a**) ε_int_ = 1 or (**b**) ε_int_ = 4, or (**c**) the MM-GBSA method using the modified OBC model^67^. Thick, opaque lines in the time courses represent the data smoothed with a Savitzky-Golay filter (window length = 51, degree of the smoothing polynomial = 2); thin, transparent lines show the unsmoothed data. Thick, opaque lines in the probability distributions show the distribution calculated with a Gaussian core density estimator (bandwidth calculated using Scott’s rule^72^); thin, transparent lines show the underlying data as a histogram. (**d**) Time-averaged mean Δ*G*_eff_, calculated using either the MM-PBSA method and an internal dielectric constant of ε_int_ = 1 or ε_int_ = 4, or the MM-GBSA method using the modified OBC model^67^. In panels a-c, data for MD simulation S_9_ is not shown because here, tauro-CDC unbinds after 225 ns (c.f. Supplementary Fig. 3b), and the corresponding data was excluded from statistical analysis.

The effective binding energy computed by MM/GBSA for tauro-CDC/GluN2D_LBD_ (mean: −33.53 ± 0.67 kcal·mol^−1^) was significantly less negative than for tauro-CDC/GluN3B_LBD_ (mean: −48.90 ± 0.62 kcal·mol^−1^) (*t*(17) = 16.84, *p* < 10^−4^) (Fig. 9d). The same trend was obtained with the MM/PBSA approach (tauro-CDC/GluN2D_LBD_: mean: −27.14 ± 0.52 kcal·mol^−1^; tauro-CDC/GluN3B_LBD_: mean: −30.54 ± 0.63 kcal·mol^−1^; *t*(17) = 4.07, *p* = 8·10^−4^). The trend was stable irrespective of using a dielectric constant of the solute of *ε* = 1 (values above; Fig. 9a,d) or 4 (tauro-CDC/GluN2D_LBD_: mean: −43.98 ± 0.24 kcal·mol^−1^; tauro-CDC/GluN3B_LBD_: mean: −45.45 ± 0.22 kcal·mol^−1^; *t*(17) = 4.49, *p* = 3·10^−4^) (Fig. 9d); higher dielectric constants have been used before to account for highly charged binding sites^28^, as given in the case of the LBDs.

To conclude, both approaches revealed more negative effective energies of tauro-CDC binding to GluN3B_LBD_ than to GluN2D_LBD_. Thus, they indicate a stronger binding of tauro-CDC to GluN3B_LBD_.

### Identification of an alternative binding site in the GluN1_LBD_/GluN2D_LBD_ interface

**Figure 10:**
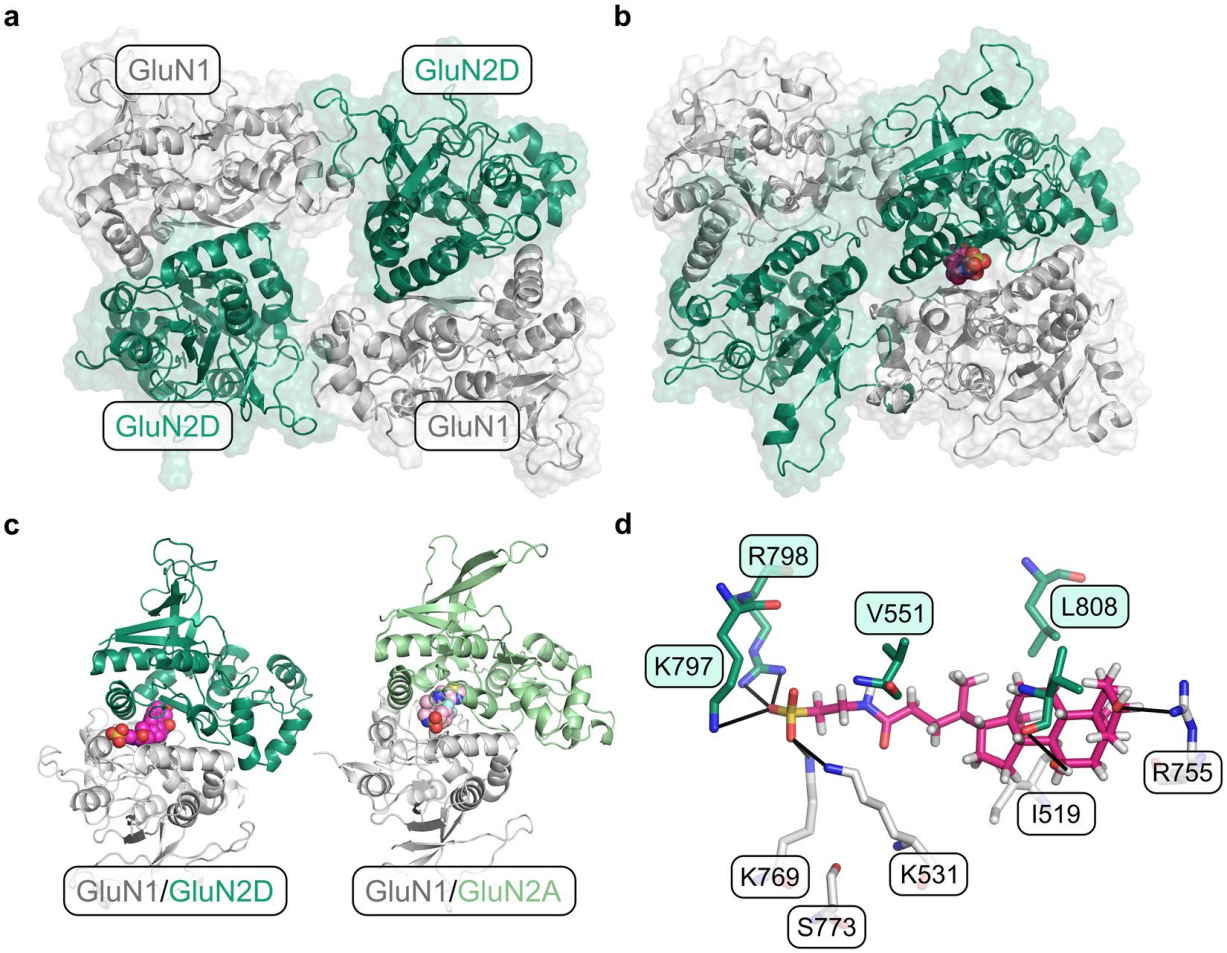
Identification of a potentially allosteric binding site in the GluN1_LBD_/GluN2_LBD_ interface. The GluN1 subunit is depicted in grey, the GluN2D subunit in dark green. (**a**) Initial model of the GluN1_LBD_/GluN2_LBD_ tetramer. (**b**) Structure of the GluN1_LBD_/GluN2_LBD_ tetramer in complex with tauro-CDC (magenta spheres). The ligand was docked to the pocket conformation that showed the highest mean local hydrophobic density^29,30^ during 500 ns of accelerated MD. (**c**) Structural comparison of the tauro-CDC-bound GluN1_LBD_/GluN2D_LBD_ interface (left) and the crystal structure of the MPX-007-bound GluN1_LBD_/GluN2A_LBD_ interface (right, PBD-ID: 5JTY^31^). The ligands are depicted as magenta and pink spheres, respectively. (**d**) Binding mode of tauro-CDC (magenta sticks) in the alternative binding pocket. Amino acids that belong to the GluN1 or GluN2D subunit are shown as white or green sticks, respectively. Possible hydrogen bonds and salt bridges are depicted as black lines.

We further aimed to identify an alternative binding mode of tauro-CDC to a potentially allosteric site in the GluN1_LBD_/GluN2D_LBD_ tetramer. To do so, we first detected potential binding pockets in a conformational ensemble obtained from 500 ns of accelerated MD simulations of the tetrameric GluN1_LBD_/GluN2D_LBD_ interface (Fig. 10a) using the MDpocket software^29^. A single binding pocket at the GluN1/GluN2D_LBD_ interface was identified, and tauro-CDC was docked into the receptor conformation in which the pocket showed the highest mean local hydrophobic density^29,30^ (Fig. 10b). The identified pocket largely overlaps with the allosteric binding pocket of the negative allosteric modulator MPX-007 in the GluN1/GluN2A interface^31^.

During 500 ns of conventional MD simulations, tauro-CDC displayed an average RMSD value of 1.90 ± 0.01 Å (2.17 ± 0.01 Å without fitting, calculated on the last 250 ns of the simulation), indicating a low structural variability of the predicted binding mode. The binding mode is stabilized by interactions of the acid moiety with the positively charged residues Lys531 and Lys769 in the GluN1 subunit and Lys797 and Arg798 in the GluN2D subunit (Fig. 10d); Leu808 in the GluN2D subunit makes a hydrophobic contact with the cholane scaffold (Fig. 10d). Functional data on GluN1/GluN2A NMDARs indicates that Phe754 in GluN1 and Val783 in GluN2A form a molecular switch that mediates allosteric inhibition^31^. The corresponding Leu808 in GluN2D may assume a similar role as Val783 in GluN2A.

These tetramer simulations suggest that the interface between the GluN1 and GluN2D subunit forms a transient pocket, to which tauro-CDC can bind.

### Point mutants

In order to verify the interaction of tauro-CDC with residues of the GluN3B LBD, Arg538 would be the primary residue for mutation studies due to its pronounced involvement in hydrogen bonds with the bile salt (Fig. 8b). However, we did not mutate Arg538 because this residue is reported to be important for agonist binding^32^. Rather, we mutated Asp447, Tyr505, and Ser701 (Fig. 8b) that also engage in hydrogen bonds with the bile salt. Yet, as these interactions are transient and, hence, likely rather weak, a decrease in the extent of tauro-CDC block would indicate an interaction of the bile salt with the mutated residue, while no effect would not exclude such an interaction due to potential enthalpy-entropy compensation effects^33^.

Mutation of Asp447 to Ala or Arg had neither an effect on agonist-induced current amplitudes (n = 2-3 dose-response curves) nor on the extent of tauro-CDC block in comparison to wild type receptor block (100 µM tauro-CDC, n = 9–12 at 10 µM Gly, n = 9–10 at 5 µM Gly). Mutation of Tyr505 to an Ala resulted in GluN3B receptors that could not be activated by its agonist glycine anymore (n = 9). Mutation of Ser701 to Ala strongly reduced glycine-induced currents from 835 ± 85 nA (n = 30) to 106 ± 12 nA (n = 29). While we observed an increase of 15% in CDC and tauro-CDC block (both 100 µM) for this mutant (n = 11–13), which might indicate an influence on CDC and tauro-CDC binding, it may also well be due to reduced glycine binding.

To conclude, none of the mutants showed a significant influence on tauro-CDC block such that no further information about binding (or non-binding) of tauro-CDC to GluN3B LBD may be inferred.

## Discussion

Here, we have shown that the bile salt CDC effectively inhibits NMDARs with a preference on GluN2D and GluN3B containing receptors. Neither did conjugation of CDC *per se* nor the type of conjugated amino salt – taurine or glycine – change the overall inhibitory potency of the bile salt or shift its subunit preference. Based on detailed pharmacological analyses and supported by MD experiments, we infer from our data that CDC inhibits NMDARs by different mechanisms that are allosteric inhibition of GluN2D and competitive block of GluN3B containing receptors.

The observed subtype preference of NMDAR inhibition by CDC is in good agreement with the one reported for structurally similar neurosteroids. Thus, pregnenolone sulfate has been shown to significantly potentiate GluN2A and GluN2B containing NMDARs but has much lower efficacy on GluN2C and GluN2D receptors^6–9^. Also, the apparent insignificance of amino acid conjugates in the interaction of bile salts with receptors is paralleled in CDC-mediated activation of the bile acid receptor TGR5, which does not discriminate between conjugation substrates^34^. Intriguingly, however, our pharmacological experiments indicated different modes of inhibition for different GluN subtypes by CDC: allosteric inhibition of GluN2D and competitive block of GluN3B. In line with this suggestion, MD simulations predicted less structural stability for tauro-CDC binding in the orthosteric pocket of GluN2D_LBD_ than in the one of GluN3B_LBD_. Effective binding energy computations comparing tauro-CDC/GluN2D_LBD_ with tauro-CDC/GluN3B_LBD_ complexes performed by two different approaches consistently revealed more negative effective binding energies of tauro-CDC binding to GluN3B_LBD_ than to GluN2D_LBD_, thus supporting stronger binding of tauro-CDC to GluN3B_LBD_.

Previous studies have shown that inclusion of configurational entropy is crucial for calculating *absolute* binding free energies^35,36^. In the present study, however, we were rather interested in *relative* binding free energies and, thus, decided to neglect contributions due to changes in the configurational entropy of the ligand or the receptor upon complex formation in order to avoid introducing additional uncertainty in the computations^35,37,38^. Finally, MD simulations of the GluN1_LBD_/GluN2D_LBD_ tetramer suggested that the interface between the GluN1 and GluN2D subunit forms a transient pocket, to which tauro-CDC can bind, which may explain the allosteric mechanism of action in this GluN subtype. We selected this interfacial pocket based on a combined approach of geometry-based cavity detection and druggability prediction that has been shown to be able to successfully identify binding-competent pocket conformations from an MD ensemble^29^. Notably, the detected pocket largely overlaps with the binding site of the negative allosteric modulator MPX-007 in the GluN1/GluN2A interface^29^. In order to verify binding of tauro-CDC to the orthosteric pocket of GluN3B_LBD_ but not GluN2D_LBD_, we quantified hydrogen bond formation between tauro-CDC and neighboring residues based on geometric criteria over the course of the MD simulations. The results show that hydrogen bonding occurred less frequently between tauro-CDC and the GluN2D_LBD_ than between tauro-CDC and the GluN3B_LBD_, in agreement with the above structural and energetic analyses of MD simulations. The tauro-CDC/GluN3B_LBD_ complex is predominantly stabilized by a hydrogen bond to Arg538 and, to a lesser extent, Asp447. Of the residues mutated to alanine in the GluN3B_LBD_, positions 505 and 701 resulted in GluN3B receptors that could either not or hardly be activated by glycine anymore. For Ser701, it was reported that it forms hydrogen bonds with the glycine carboxylate group^32^, so it is not surprising that mutation of this site affected channel function. Mutation of Asp447 to an Ala or Arg had neither an effect on agonist-induced current amplitudes nor on the extent of tauro-CDC block, which does not exclude that Asp447 interacts with tauro-CDC, as enthalpy-entropy compensation effects upon mutation may account for the loss of interaction in the mutant^33^. In particular, we speculate that the Asp447Ala mutation leads to a loss in binding enthalpy upon disruption of the tauro-CDC-4Asp47 hydrogen bond, but this loss in binding enthalpy could be compensated by both a gain in entropy of the ligand due to an increase in translational and rotational degrees of freedom and a gain in entropy of the receptor due to increased conformational freedom of the residues surrounding the mutation site. The latter effect might especially be relevant due to the stabilizing interaction between Asp447 and Arg474, which would break upon mutation of Asp447 to alanine. Similarly, in the Asp447Arg mutant, the interaction between tauro-CDC and receptor could be preserved if arginine acted as a hydrogen bond donor for the 3-OH group of tauro-CDC, which would in turn act as a hydrogen bond acceptor. Moreover, repulsive forces between Arg447 in the Arg447Arg mutant and Arg474 could lead to a gain in receptor entropy.

The inhibition of GluNs by bile salts required concentrations that are hardly reached in the CNS, where CDC and cholate exists in the nmol/g range^39,40^. However, NMDARs are also widely expressed in non-neural peripheral tissues. These receptors have many distinct physiological and pathophysiological roles, and there is evidence that peripheral NMDARs may use alternative agonists such as L-HCA and QN^5^. We could show that tauro-CDC also reduced currents elicited by these alternative NMDAR agonists, indicating potential modulation of peripheral NMDAR function by bile salts. Whereas under physiological conditions only low µM amounts of bile salts are present in serum and urine, in cholestatic disease states like inherited progressive cholestasis, severe forms of intrahepatic cholestasis of pregnancy or acute hepatitis, plasma concentrations may rise to hundred µM and more^10–12^. As blood cells including platelets express NMDARs, it may well be hypothesized that bile salt mediated receptor inhibition contributes to the reported platelet activation defects and impaired thrombus formation in cholestatic liver disease^41^. Despite the observed preference of bile salts for specific receptor isoforms, it remains difficult to predict the exact physiological or pathophysiological consequences of their action on NMDAR signaling. The net effect of bile salts on NMDARs will eventually depend on the overall expression, stoichiometric assembly, and posttranscriptional and posttranslational modifications of individual GluN isoforms in a given cell type^42^. On a pharmacological perspective, however, the distinct modes of NMDAR inhibition by bile salts, i.e. allosteric versus competitive, may be exploited for developing targeted interference strategies holding isoform specificity.

## Materials and Methods

### Molecular biology

The cDNAs coding for NMDA and kainate receptors, subcloned into pSGEM for expression in *Xenopus laevis* oocytes, were kindly provided by Michael Hollmann (Ruhr University Bochum). AMPA receptor cDNAs were inserted in pGEM-HE for cRNA synthesis. The following cDNAs were used: GluN1-1a (U08261), GluN2A (NM_012573), GluN2B (NM_012574), GluN2D (U08260), GluN3B (NM_130455), GluK2 (NM_019309), GluA1 (NM_031608) and GluA2 (M38061). These cDNAs are from rat except for GluN3B, which is from mouse. Point mutants were generated by polymerase chain reaction (PCR)-directed mutagenesis with specific mismatch primers (biomers.net). cRNA was synthesized from 1 µg of linearized plasmid DNA using an *in vitro* transcription kit (mMESSAGE mMACHINE® T7 Transcription Kit, Thermo Fisher Scientific).

### Heterologous expression of iGluRs in Xenopus laevis oocytes

Oocytes were obtained from parts of the ovaries surgically removed from *Xenopus laevis* in accordance with German law and approved by the local authorities of the Heinrich Heine University of Düsseldorf. Lumps of around 20 oocytes were incubated with 10 mg/ml collagenase type 2 (Worthington Biochemical Corporation) for 1–1.5 h in Ca^2+^-free Barth’s solution (in mM: 88 NaCl, 1 KCl, 2.4 NaHCO_3_, 0.82 MgSO_4_, 20 HEPES, pH adjusted to 7.5 with NaOH) with slow agitation to remove the follicular cell layer and then washed extensively with Barth’s solution containing Ca^2+^ (in mM: 88 NaCl, 1 KCl, 2.4 NaHCO_3_, 0.82 MgSO_4_, 0.41 CaCl_2_, 0.33 Ca(NO_3_)_2_, 20 HEPES, pH adjusted to 7.5 with NaOH) to inactivate the collagenase. Oocytes were maintained in Barth’s solution charged with antibiotic and antimycotic solution (containing penicillin, streptomycin and amphotericin B, Sigma-Aldrich) at 18 °C. Alternatively, *Xenopus laevis* oocytes were purchased from EcoCyte Bioscience (Castrop-Rauxel, Germany). Within 24 h after surgery, oocytes of stages V-VI were injected with 3.8 ng GluN1 and 5 ng GluN2A or GluN2B cRNAs, 7.6 ng GluN1 and 10 ng GluN2D cRNA or 7.6 ng GluN1 and 6.4 ng GluN3B-cRNA per oocyte (molecular GluN1 to GluN2 ratio: 1:1; GluN1 to GluN3 ratio: 1:1) using a Micro4 nanoliter injector (World Precision Instruments, Sarasota, FL, USA).

### Electrophysiological recordings

Two-electrode voltage clamp recordings of oocyte current responses were performed 3–5 days after cRNA injection at −70 mV holding potential with a Turbo Tec-03X amplifier (npi electronic, Germany) controlled by Pulse software (HEKA, Germany). Electrodes were filled with 3 M KCl and had resistances of 0.5–1.5 MΩ. Oocytes were continuously superfused with calcium-free Ba^2+^-Ringer’s solution (in mM: 115 NaCl, 2.5 KCl, 1.8 BaCl_2_ and 10 HEPES, pH 7.2) to prevent the activation of endogenous Ca^2+^-gated chloride channels. Agonists and bile salts were applied for 20–40 s by superfusion. Agonist and bile salt blocker potencies were determined by recording current responses induced by the application of increasing agonist concentrations or increasing bile salt concentration to the same oocyte. Agonist-induced currents were normalized to the maximum response under saturating agonist concentration. In case of inhibition by bile salts the relative reduction in agonist-induced current was determined. Data from each oocyte was fitted separately to the Hill equation using Origin 9 software. The resulting EC_50_ or IC_50_ values were averaged. Current-voltage relationships were determined by ramping the holding potential from −140 mV to +50 mV corrected for background conductivities. Data are reported as mean ± standard error of the mean (SEM). Statistical significance was determined using the unpaired, two-tailed Student’s t-test.

### Molecular modeling and molecular dynamics simulations

#### Preparation of tauro-CDC/GluN2D_LBD_ and tauro-CDC/GluN3B_LBD_ complex structures

In order to generate full-length structures of rat GluN2D and mouse GluN3B LBDs (from now on referred to as GluN2D_LBD_ and GluN3B_LBD_), we first generated a multiple sequence alignment of all rat and mouse GluN1, GluN2, and GluN3 subunit sequences using the MAFFT^43^ server with default settings applied. The alignment was further refined using the GLProbs software^44^ with two passes of consistency transformation and 100 passes of iterative refinement. The resulting alignment was then used as a template to manually align the sequence portions resolved in the selected template structures for GluN2D_LBD_ (PDB ID: 3OEK^45^) and GluN3B_LBD_ (PDB ID: 2RCA^32^) to the target sequences using the BioLuminate package (Schrödinger Release 2018–3: BioLuminate, Schrödinger, LLC, New York, NY, 2018) of the Schrödinger Suite (Small-Molecule Drug Discovery Suite 2018-3, Schrödinger, LLC, New York, NY, 2018). This procedure was necessary to account for insertions and deletions present in the template structures (Supplementary Fig. 1). Structural modeling was also performed with the BioLuminate package using the energy-based model building method (Schrödinger Release 2018-3: Prime, Schrödinger, LLC, New York, NY, 2018). For identical residues in the alignment, side chain rotamers were retained, whereas for non-identical residues, side chain and main chain atoms were energy-minimized using the Prime module (Schrödinger Release 2018-3: Prime, Schrödinger, LLC, New York, NY, 2018) employing the VSGB solvation model^46^ and the OPLS3 force field^47^; all other settings were kept at their default values.

To generate atomic charges for tauro-CDC, we first generated a three-dimensional molecular structure of tauro-CDC in the Maestro interface (Schrödinger Release 2018-3: Maestro, Schrödinger, LLC, New York, NY, 2018) of the Schrödinger Suite. Further preparation of the structure in LigPrep (Schrödinger Release 2018-3: LigPrep, Schrödinger, LLC, New York, NY, 2018) (default settings) returned a single state with a negatively charged sulfonate moiety. Conformers of this structure were then generated in the MacroModel module (Schrödinger Release 2018-3: MacroModel, Schrödinger, LLC, New York, NY, 2018) using the Monte-Carlo Multiple Minimum search algorithm^48^, keeping all other settings at their default values. The conformer with the lowest potential energy that did not display intramolecular hydrogen bonding was subjected to quantum mechanical geometry optimization at the HF/6-31 G(d) level of theory in the GAUSSIAN 09 software (Revision A.02)^49^. A tight convergence criterion for the self-consistent field iteration process was set, and computation of electrostatic potential points with a density of ~1.68 pt/au^2^ (6 pt/Å^2^) was invoked. The geometry-optimized structure was visually inspected for the absence of intramolecular hydrogen bonds and subjected to the standard RESP procedure^50,51^ as implemented in the Antechamber set of programs to obtain the atomic point charges.

Binding modes for tauro-CDC in GluN2D_LBD_ and GluN3B_LBD_ were generated using the standard Induced Fit protocol implemented in the Schrödinger Suite^25^. The docking grid was centered on the coordinates of the bound ligand in the GluN2D and GluN3B template structure, respectively. In the initial Glide docking stage, the van der Waals radii of the receptor and ligand atoms were reduced to half their initial value, and a maximum of 20 poses were allowed to be carried over to the Prime (Schrödinger Release 2018-3: Prime, Schrödinger, LLC, New York, NY, 2018) refinement stage. For each generated pose, the receptor residues within 5 Å were energy-minimized and their side chain rotamers optimized. During the final Glide (Schrödinger Release 2018-3: Glide, Schrödinger, LLC, New York, NY, 2018)^52,53^ docking stage, tauro-CDC was redocked into all receptor conformers using XP precision^54^. The tauro-CDC/GluN2D_LBD_ and tauro-CDC/GluN3B_LBD_ complexes with the lowest IFD score^25^ were considered for all subsequent calculations.

#### Molecular dynamics simulations of tauro-CDC/GluN2D_LBD_ and tauro-CDC/GluN3B_LBD_ complexes

All molecular dynamics (MD) simulations were performed using the mixed single precision/fixed precision GPU (CUDA) version of PMEMD^55^ in the Amber14 suite of molecular simulation programs^56^. Hydrogen mass repartitioning^57^ was employed to enable a time step of 4 fs for integration. The Langevin thermostat^58^ with a collision frequency of 0.01 ps^−1^ and a target temperature of *T* = 300 K was used for temperature control. Covalent bonds involving hydrogen atoms were constrained using the SHAKE algorithm^59^. Long-range electrostatic interactions were estimated using the Particle Mesh Ewald method^60^, and a cutoff of 8 Å was used for short-range electrostatics and van der Waals forces.

All following steps were performed 10 times for each of the two complexes (tauro-CDC/GluN2D_LBD_ and tauro-CDC/GluN3B_LBD_) so as to obtain 10 independent simulations per complex. The initial structures were minimized using a three-step procedure. First, the coordinates of all solute molecules were restrained by a harmonic potential with a force constant of 2.0 kcal mol^−1^ Å^−2^ while 2,000 steps of steepest descent minimization followed by 3,000 steps of conjugate gradient minimization were carried out. This step was repeated with the restraints switched from the solute to the solvent molecules. During the following final minimization step, the restraints were removed, and 3,000 steps of steepest descent minimization followed by 7,000 steps of conjugate gradient minimization were performed. 20 ps of NVT-MD (the solute was restrained with a force constant of 2.0 kcal mol^−1^ Å^−2^) were carried out while heating the system from 0 K to 300 K, followed by additional 5 ps of NVT-MD at 300 K. Density adaptation of the system was achieved by 75 ps of NPT-MD (solute restrained, force constant: 2.0 kcal mol^−1^ Å^−2^). An additional 1.7 ns of restrained NPT-MD were carried out before switching to the NVT ensemble. Here, 3.2 ns of restrained MD were performed prior to the start of the production phase, with harmonic restraints (force constant: 2 kcal mol^−1^ Å^−2^) applied to only those C_α_ atoms that are closest to the center of mass of a secondary structure element (α-helix or β-sheet). The subsequent production phase consisted of 500 ns of NVT-MD (restraints as in the final NVT step of the equilibration phase), resulting in an aggregate simulation time of 5 μs per complex (10 μs in total). Coordinates for analysis and post-processing were saved every 20 ps. Post-processing and analysis of the MD trajectories was performed in CPPTRAJ^61^ as implemented in AmberTools15. Trajectories were visually inspected with VMD^62^. The two-tailed Student’s *t*-test was used to determine statistically significant differences in time-averaged quantities between GluN2D and GluN3B.

#### Calculation of effective binding energies

Effective binding energies (Δ*G*_eff_) of tauro-CDC to GluN2D_LBD_ and GluN3B_LBD_ were calculated using the MM/PBSA and MM/GBSA approaches as implemented in the MMPBSA.py module^63^ in AmberTools15^56^. All calculations were performed on 500 snapshots per trajectory extracted at regular intervals of 1 ns, corresponding to a total of 5,000 snapshots per approach for both the tauro-CDC/GluN2D_LBD_ complex and the tauro-CDC/GluN3B_LBD_ complex. The MM/PBSA calculations were performed with values of 1 and 4 for the internal dielectric constant (ε_int_), to test for robustness of the relative Δ*G*_eff_ with respect to this parameter^28^. The polar part of the contribution to the solvation free energy was calculated by the linear Poisson-Boltzmann equation using an ionic strength of 100 mM and an external dielectric constant (ε_ext_) of 80. Solutions for the linear PB equation were computed with 1,000 iterations on a cubic grid with 0.5 Å spacing between grid points. The nonpolar part of the contribution to the solvation free energy was considered proportional to the solvent accessible surface area (SASA) and computed with the equation Δ*G*_nonpol,solv_ = *γ*(SASA) + *β*, where *γ* and *β* are parameterized constants^64^. The SASA was calculated with a solvent probe radius of 1.4 Å, Tan & Luo radii^65^ for the protein, and mbondi radii^66^ for the ligands. The MM/GBSA calculations were performed using the modified OBC model (GB^OBC^II)^67^, an ionic strength of 100 mM and default values for γ (0.005 kcal mol^−1^ Å^−2^) and β (0.0 kcal mol^−1^).

Results are expressed as Δ*G*_eff_ ± standard error of the mean (SEM), where Δ*G*_eff_ is the average effective binding energy over all snapshots and the overall SEM is estimated as the SEM of the single trajectories propagated over all trajectories. The two-tailed Student’s *t*-test was used to determine statistically significant differences in Δ*G*_eff_ between GluN2D and GluN3B.

#### Preparation of GluN1_LBD_/GluN2D_LBD_ tetramer structures

The structure of the rat GluN1 LBD (GluN1_LBD_) was prepared analogously to the structure of GluN2D_LBD_ (see above for further details), using the crystal structure of the rat GluN1 LBD (PDB ID: 1PB7^68^) as a template to which only missing residues and loops were added during the modeling step. To construct the LBD tetramer from the individual LBD structures, the coordinates of the respective LBD monomers were superimposed onto their corresponding monomer units in the structure of the GluN1/GluN2D NMDA receptor (PDB ID: 5FXH^69^) (Fig. 10a).

#### Accelerated molecular dynamics simulation of the GluN1_LBD_/GluN2D_LBD_ tetramer

A single, 500 ns long accelerated MD (aMD) simulation^70^ of the GluN1_LBD_/GluN2D_LBD_ tetramer was prepared, heated, and equilibrated analogously to the classical MD simulations of the tauro-CDC/GluN2D_LBD_ and tauro-CDC/GluN3B_LBD_ complex (see above). During the production phase, bias parameters of α_D_ = 781.0 kcal mol^−1^ and α_P_ = 50,035.0 kcal mol^−1^, a total dihedral energy threshold of E_threshD_ = 18,950.0 kcal mol^−1^, and a total potential energy threshold of E_threshP_ = −341,718.0 kcal mol^−1^ were set on the basis of empirical formulas that were previously used to estimate these parameters^70,71^.

#### Preparation of tauro-CDC//GluN1_LBD_/GluN2D_LBD_ complex structures

To identify a possible allosteric binding site in the GluN1_LBD_/GluN2D_LBD_ tetramer, the MDpocket software^29^ was applied on all 25,000 snapshots of the 500 ns long aMD trajectory (see above). A binding pocket at the GluN1/GluN2D_LBD_ interface was identified into which tauro-CDC was docked, using the snapshot in which the selected pocket displayed the highest mean local hydrophobic density^29,30^ (Supplementary Fig. 2).

#### Molecular dynamics simulations of the tauro-CDC//GluN1_LBD_/GluN2D_LBD_ complex

A single 500 ns long conventional MD simulation of the tauro-CDC//GluN1_LBD_/GluN2D_LBD_ tetramer complex was generated and analyzed analogously to the classical MD simulations of the tauro-CDC/GluN2D_LBD_ and tauro-CDC/GluN3B_LBD_ complex (see above).

## Supplementary information

Supplementary Information
