# Supplementary material for: Isoform-specific Inhibition of N-methyl-D-aspartate Receptors by Bile Salts
Source: Sci Rep. 2019 Jul 11;9:10068. doi: 10.1038/s41598-019-46496-y (PMC6624251; doi:10.1038/s41598-019-46496-y)
Supplement: Supplementary file 1 — Supplementary Information [file 41598_2019_46496_MOESM1_ESM.docx]

# Supplementary Information

# Isoform-specific Inhibition of *N*-methyl-D-aspartate Receptors by Bile Salts

Angela Koch^a,§^, Michele Bonus^b,§^, Holger Gohlke^b,c^, Nikolaj Klöcker^a,*^

*^a^ Institute of Neural and Sensory Physiology, Medical Faculty, Heinrich Heine University Düsseldorf, 40225 Düsseldorf, Germany*

*^b^ Institute for Pharmaceutical and Medicinal Chemistry, Heinrich Heine University Düsseldorf, 40225 Düsseldorf, Germany*

*^c^ John von Neumann Institute for Computing (NIC), Jülich Supercomputing Centre (JSC) & Institute for Complex Systems - Structural Biochemistry (ICS 6), Forschungszentrum Jülich GmbH, 52425 Jülich, Germany*

*§ these authors contributed equally*

**Correspondence to: Nikolaj Klöcker, Institute of Neural and Sensory Physiology, Medical Faculty, Heinrich Heine University, Universitätsstraße 1, 40225 Düsseldorf, Germany, Tel.: 0049-211-81-12687; Fax: 0049-211-81-14231; E-mail: nikolaj.kloecker@uni-duesseldorf.de*

# Supplementary Figures


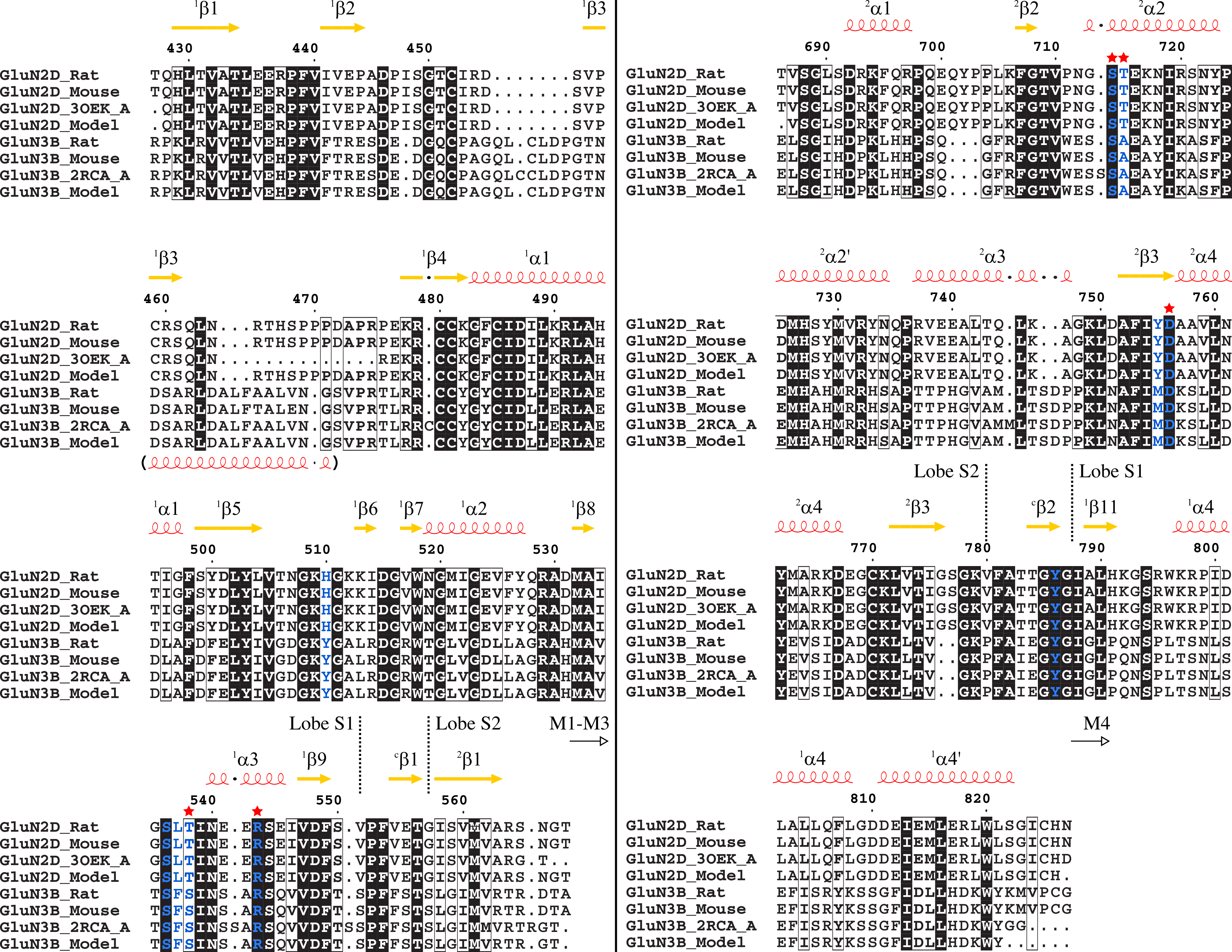


**Supplementary Figure S1.** Sequence alignment of the rat and mouse GluN2D_LBD_ and GluN3B_LBD_ sequences and the sequences of the respective template structures and the final models. α helices and β sheets are shown in red and yellow, respectively, and numbered sequentially in each of the two structural lobes S1 and S2. The left part of the figure shows the portion of the LBD sequence that precedes transmembrane segments M1-M3, the right part the portion of the LBD sequence that follows these segments.


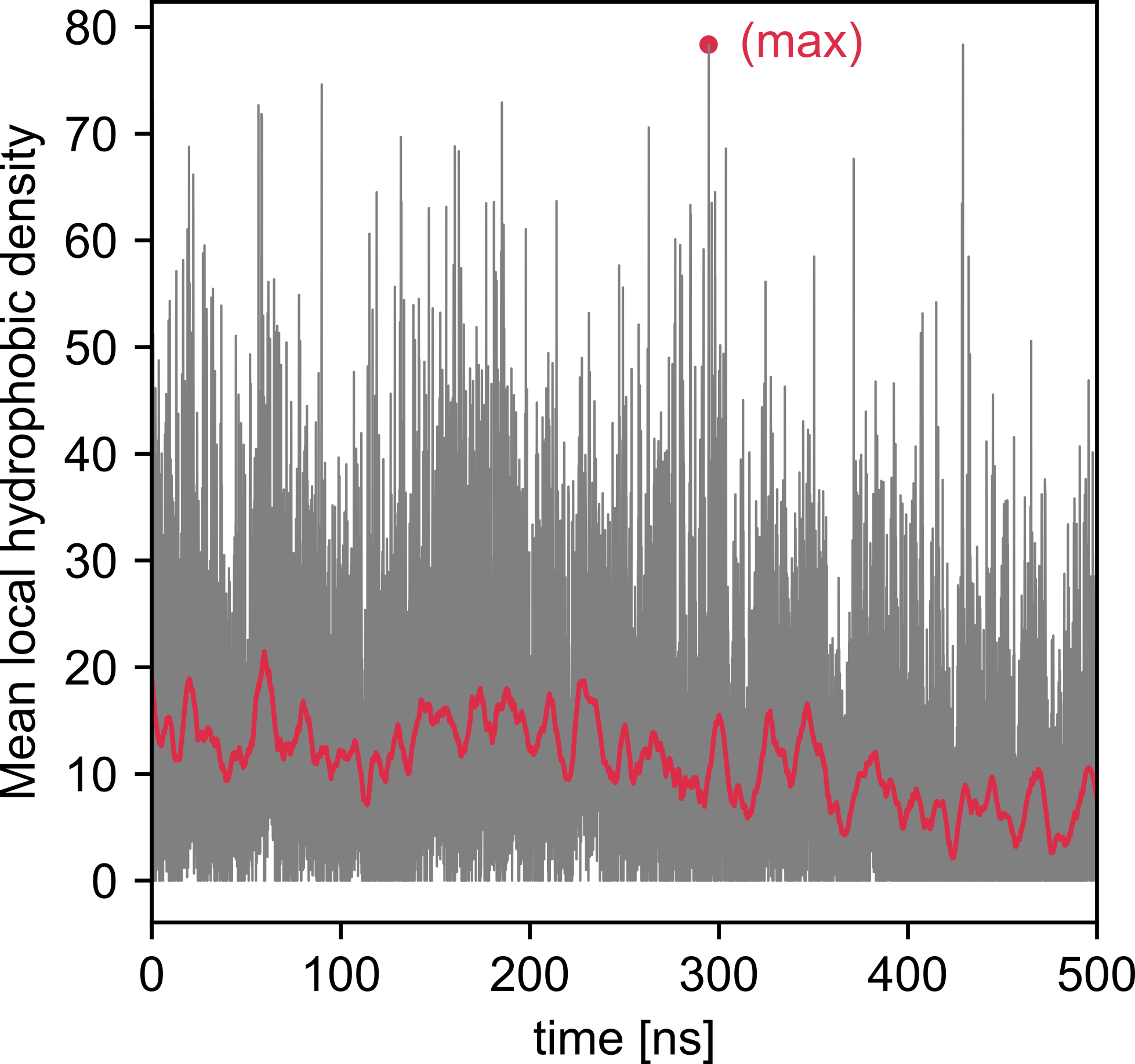


**Supplementary Figure S2.** Time course of the mean local hydrophobic density (MLHD)^29,30^ for the identified pocket in the GluN1_LBD_/GluN2_LBD_ interface. The snapshot in which the pocket displayed the maximum MLHD (denoted as "max") was selected for docking of tauro-CDC.


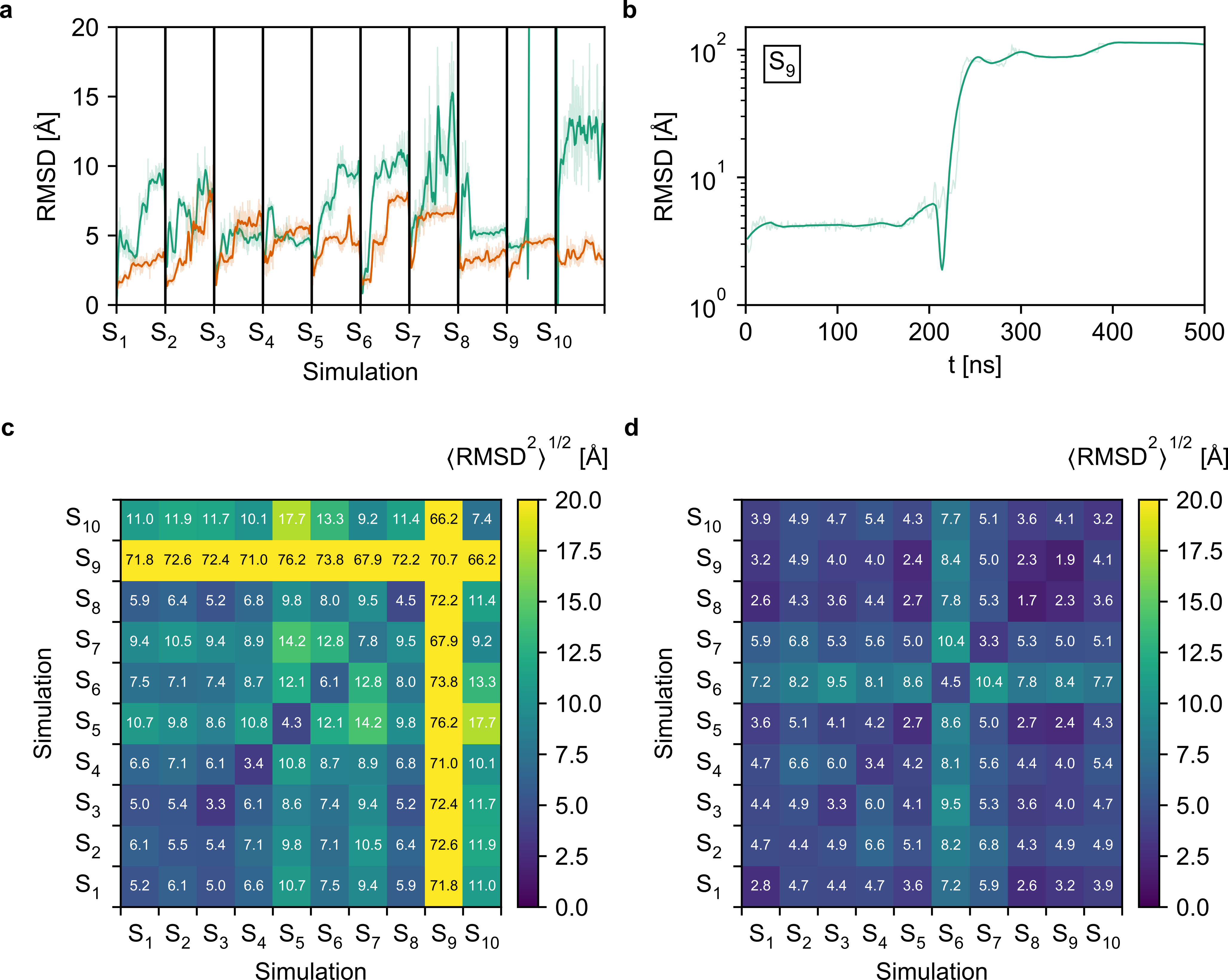


**Supplementary Figure S3.** (**a**) RMSD of tauro-CDC after least squares fitting of the C_α_ atom coordinates of the GluN2D_LDB_ (green) or GluN3B_LBD_ (orange). (**b**) RMSD of tauro-CDC during MD simulation 9 (S_9_) after least squares fitting of the C_α_ atom coordinates of the GluN2D_LDB_. (**c**) Quadratic means of the pairwise RMSDs calculated for pairs of individual simulations of the tauro-CDC/GluN2D_LBD_ complex and (**d**) the tauro-CDC/GluN3B_LBD_ complex. Coordinate fitting was performed as described in panel a and Fig. 7a, b, respectively.

# Supplementary Tables

**Table S1.** Mean (± SEM) reduction of agonist-induced NMDA receptor currents

| **Bile salt** | **Reduction of *I*_Glu/Gly_ by bile salt [%] (*n*)** | | | |
| --- | --- | --- | --- | --- |
|  | GluN2A | GluN2B | GluN2D | GluN3B |
| Cholate | 23 ± 4 (10) | 7 ± 2 (11) | 12 ± 1 (11) | 20 ± 3 (6) |
| Glycocholate | 8 ± 3 (9) | 3 ± 1 (8) | 10 ± 1 (8) | 14 ± 1 (6) |
| Taurocholate | 7 ± 3 (9) | 7 ± 2 (7) | 9 ± 2 (8) | 16 ± 1 (6) |
| CDC | 7 ± 2 (10) | 13 ± 1 (13) | 37 ± 2 (14) | 58 ± 5 (12) |
| Glyco-CDC | 11 ± 3 (9) | 17 ± 2 (9) | 34 ± 2 (9) | 50 ± 4 (8) |
| Tauro-CDC | 8 ± 2 (8) | 15 ± 2 (9) | 33 ± 2 (14) | 47 ± 3 (12) |

Mean block by 100 µM bile salt of currents induced by 150 µM glutamate and 10 µM glycine. *n*, number of oocytes.

**Table S2.** Mean IC_50_ or EC_50_ values for Tauro-CDC and agonists (± SEM) on GluN2D

| **Condition** | **IC_50 Tauro-CDC_ [µM] (*n*)** | **max. block by Tauro-CDC [%] (*n*)** |
| --- | --- | --- |
| 10 µM Gly, 150 µM Glu | 179 ± 11 (13) | 87 ± 3 (13) |
| 10 µM Gly, 0.51 µM Glu | 137 ± 8 (11) | 100 ± 4 (11) |
| 0.13 µM Gly, 150 µM Glu | 153 ± 8 (8) | 98 ± 3 (8) |
|  | EC_50_ **_Glu_** [µM] (*n*) | max. Glu-induced current [normalized] (*n*) |
| 10 µM Gly | 1.03 ± 0.12 (7) | 1.00 ± 0.17 (7) |
| 10 µM Gly  + 180 µM Tauro-CDC | 0.82 ± 0.15 (7) | 0.25 ± 0.06 (7) |
|  | EC_50_ **_Gly_** [µM] (*n*) | max. Gly-induced current [normalized] (*n*) |
| 150 µM Glu | 0.25 ± 0.05 (12) | 1.00 ± 0.09 (12) |
| 150 µM Glu  + 180 µM Tauro-CDC | 0.15 ± 0.03 (13) | 0.48 ± 0.07 (13) |
